# Diversity of culturable bacteria endowed with antifungal metabolites biosynthetic characteristics associated with tea rhizosphere soil of Assam, India

**DOI:** 10.1186/s12866-021-02278-z

**Published:** 2021-07-18

**Authors:** Jintu Dutta, Debajit Thakur

**Affiliations:** 1 Microbial Biotechnology Laboratory, Life Sciences Division, Institute of Advanced Study in Science and Technology, Guwahati, Assam India; 2 Present Address: Centre for the Environment, Indian Institute of Technology, Guwahati, Assam India

**Keywords:** Antagonist activity, ARDRA, BOX-PCR, Chitinase, Rhizosphere

## Abstract

**Background:**

Rhizosphere soil is a crucial niche for the diverse beneficial microbial communities in plant-microbe interactions. This study explores the antagonistic potential and diversity of the rhizosphere soil bacteria from commercial tea estates of Assam, India which comes under the Indo-Burma mega-biodiversity hotspot. Rhizosphere soil samples were collected from six different tea estates to isolate the bacteria. The bacterial isolates were subjected to evaluate for the antagonistic activity against fungal pathogens. The potential isolates were investigated for chitinase production and the presence of chitinase gene. The bacterial genetic diversity was studied by Amplified Ribosomal DNA Restriction Analysis (ARDRA) and BOX-PCR fingerprinting.

**Results:**

A total of 217 rhizobacteria were isolated from tea rhizosphere soil, out of which 50 isolates exhibited the potential antagonistic activity against fungal pathogens. Among them, 12 isolates showed extracellular chitinase activity and the presence of chitinase genes. The chitinase genes were sequenced and the analysis of the sequences was performed by using PDB protein databank at the amino acid level. It showed the presence of ChiA and ChiA74 gene in the 6 most potent isolates which are involved in the hydrolysis of chitin. These isolates also exhibited antagonistic activity against all tested fungal pathogens. The diversity of 50 antagonistic bacterial isolates were analyzed through ARDRA and BOX-PCR fingerprinting. Diversity analysis and molecular identification of the rhizosphere isolates revealed that these antagonistic isolates predominantly belonged to the genus *Bacillus* followed by *Enterobacter*, *Serratia*, *Lysinibacillus*, *Pseudomonas*, and *Burkholderia*.

**Conclusion:**

The present study establishes that rhizobacteria isolated from the poorly explored tea rhizosphere soil could be a rich reservoir for the investigation of potential antagonistic bacterial candidates for sustainable agricultural and industrial applications.

**Supplementary Information:**

The online version contains supplementary material available at 10.1186/s12866-021-02278-z.

## Background

The factors controlling the distribution and abundance of soil microorganisms are still poorly understood despite soil microbes being the dominant engines of biogeochemical cycles and a major pool of living biomass in terrestrial ecosystems [14]. A recent study conducted using a variety of molecular or biochemical approaches has started to explore the distributional patterns exhibited by soil microbial communities and biotic or abiotic factors driving these patterns [30]. Among different soil types, rhizosphere soil is considered as one of the most active regions in soil that is governed by intense interactions between plant and root-associated microbes. The microbial communities residing in the rhizosphere play a pivotal role in plant growth promotion and protection. The rhizosphere soil bacteria thrive on root exudates efficiently and therefore the population of rhizosphere microbes is found to be higher than bulk soil [34]. Moreover, it was observed that the soil microbial and enzymatic properties respond relatively quickly to small changes that occurred in soil conditions and thus microbial properties and enzymes are considered to be good indicators of soil [40]. Among the different edaphic factors, the pH of the soil is also considered as a significant factor that affects the composition and diversity of soil bacterial communities [13, 17, 30, 37]. Moreover, the molecular studies on bacterial diversity have revealed a large richness of species, which promote plant growth and yield, compete for (or inhibit) pathogens, solubilize phosphate, or contribute to nitrogen assimilation in plants [29]. This technological advance has now become so pervasive that it is being regularly applied to explore soils and plants of agricultural interest.

The microbial interaction with tea plants is one of the less explored scientific area with potential future research. Tea plants are massively cultivated in Assam of Northeast India. The climatic condition and geographic location of the region are very much favorable for tea cultivation which makes entire India a leading tea production country in the world. However, this climatic condition of Northeast India offers a congenial environment for enormous numbers of fungal pathogens and pest invasion which leads to a considerable amount of crop loss annually. Tea plants are absolutely acclimatized to warm and humid conditions of the region, and the peculiar cultural conditions make them more disease susceptible [4]. The extensive use of pesticides, fungicides and other agrochemicals in order to control the diseases and pests, is also a burden to planters as well as to the environment that can also develop resistance or execute other economically important insects [16]. Therefore, one has to be very cautious and judicial about the use of such perilous chemicals in any crop fields. The demand for sustainable chemicals free production of tea also leading to a movement toward organic tea cultivation. Therefore, the present time demands the development in novel sustainable strategies for crop protection and enhancement that do not rely on harmful chemicals. Besides, the Northeast of India is also a part of the Indo-Burma mega-biodiversity hotspot [25] where microbial communities and their functions in soil of different regions are still to be explored.

The goal of the present study was to isolate the rhizosphere soil bacteria from different commercial tea estates located in Assam, India and evaluate them for the potential antagonistic activity against some major tea fungal pathogens. The antagonistic isolates were also screened for the presence of one of the major fungal cell wall degrading enzymes, the chitinase gene within their genome. Further, the analysis of antagonistic microbial diversity present in the tea rhizosphere soil was carried out by using Amplified Ribosomal DNA Restriction Analysis (ARDRA) and BOX-PCR fingerprinting. Therefore, this study was made an effort to investigate the promising rhizosphere soil bacteria for potential antagonistic activity against a wide array of major tea fungal pathogens.

## Results

### Isolation of rhizobacteria

A total of 217 rhizobacteria were isolated from rhizosphere soil in six different commercial tea estates of Assam, India. The culturable rhizobacteria were isolated from collected soil samples by using four different isolation media. The rhizobacterial isolates were enumerated based on their distinctive colonial morphology.

### In vitro antifungal assay

All the 217 isolates were subjected for in vitro antifungal activity against six test phytopathogens to evaluate their antagonistic potential. From the assay, 50 (23%) isolates exhibited positive antifungal activity. Out of these 50 positive isolates, 34 (68%) isolates exhibited antagonistic activity against *N. sphaerica* (KJ767520), 33 (66%) isolates showed antagonistic activity against *P. theae* (ITCC 6599), 19 (38%) isolates showed antagonistic activity against *C. eragrostidis* (ITCC 6429), 27 (54%) isolates showed antagonistic activity against *G. cingulate* (MTCC 2033), 24 (48%) isolates showed antagonistic activity against *R. solani* (MTCC 4633), and 27 (54%) isolates exhibited antagonistic activity against *F. oxysporum* (MTCC 284). Furthermore, 16 (32%) isolates exhibited antifungal activity against at least four test pathogens and 6 (12%) isolates i.e., HK28, SN18, SN25, HK17, TG1 and TT19 showed potential antagonistic activity which inhibited the growth of all six test fungal pathogens (Table 1, Fig. S1).

**Table 1:** In vitro antifungal assay of rhizobacteria isolated from rhizosphere soil of different commercial tea estates of Assam, India against fungal phytopathogens

Sl No.	Strain Code	*N. sphaerica* (KJ767520)	*P. theae* (ITCC 6599)	*C. eragrostidis* (ITCC 6429)	*G. cingulata* (MTCC 2033)	*R. solani* (MTCC 4633)	*F. oxysporum* (MTCC 284)
		Growth inhibition (%)^a^					
1	KH45	28.6 ± 0.1	NA	16.6 ± 0.1	NA	25 ± 0.1	27.5 ± 0.3
2	HK21	23.6 ± 0.3	NA	18 ± 0.2	10.5 ± 0.1	22.5 ± 0.1	21.5 ± 0.3
3	SN30	NA	7.8 ± 0.2	NA	27.5 ± 0.3	NA	18.5 ± 0.1
4	TG30	8.5 ± 0.2	13.1 ± 0.1	NA	NA	NA	22.5 ± 0.2
5	TG24	34.2 ± 0.3	22 ± 0.1	NA	NA	25 ± 0.3	40 ± 0.5
6	TG27	17.9 ± 0.2	10.5 ± 0.1	NA	NA	5 ± 0.1	NA
7	HK25	NA	32.6 ± 0.1	NA	29.5 ± 0.3	NA	27.5 ± 0.5
8	DT1	NA	2.6 ± 0.1	NA	5 ± 0.1	NA	NA
9	TT6	26.1 ± 0.1	NA	NA	28.7 ± 0.2	NA	35 ± 0.1
10	KH34	20.5 ± 0.1	5.2 ± 0.1	NA	NA	NA	30 ± 0.3
11	HK54	17.7 ± 0.2	23 ± 0.1	26.6 ± 0.2	NA	20 ± 0.1	NA
12	DT15	NA	8.2 ± 0.1	NA	NA	14.5 ± 0.1	NA
13	HK27	22.8 ± 0.3	NA	NA	15 ± 0.1	NA	17.5 ± 0.2
14	KH49	22.3 ± 0.2	NA	24 ± 0.1	22 ± 0.1	NA	37.5 ± 0.5
15	SN22	25.1 ± 0.2	NA	25.1 ± 0.2	22.1 ± 0.2	18.3 ± 0.2	27.3 ± 0.3
16	HK28	25.1 ± 0.1	26.3 ± 0.3	26 ± 0.1	17 ± 0.1	20 ± 0.1	23 ± 0.1
17	HK31	NA	7.9 ± 0.1	25.6 ± 0.3	NA	21 ± 0.4	NA
18	SN27	7.1 ± 0.4	NA	NA	10 ± 0.1	NA	NA
19	SN18	28.6 ± 0.1	29.6 ± 0.1	23.3 ± 0.1	22.5 ± 0.1	25 ± 0.1	32 ± 0.1
20	HK8	13.6 ± 0.3	NA	7.6 ± 0.2	12.5 ± 0.1	NA	NA
21	SN25	31 ± 0.1	27.8 ± 0.2	26 ± 0.1	22.50.1	21 ± 0.3	26.5 ± 0.1
22	HK18	NA	13.1 ± 0.1	NA	17.5 ± 0.3	NA	22.5 ± 0.2
23	HK36	NA	18.4 ± 0.2	22 ± 0.1	20 ± 0.1	NA	27.5 ± 0.3
24	DT18	14.9 ± 0.2	NA	NA	10.5 ± 0.1	7 ± 0.1	NA
25	DT2	NA	2.6 ± 0.1	NA	NA	5.1 ± 0.2	17.5 ± 0.5
26	HK33	NA	12.6 ± 0.1	NA	15 ± 0.1	NA	NA
27	HK51	NA	5.2 ± 0.1	NA	NA	NA	15 ± 0.1
28	SN29	38.5 ± 0.2	25.2 ± 0.1	35 ± 0.4	30 ± 0.5	25 ± 0.1	NA
29	HK20	NA	12 ± 0.1	NA	NA	10 ± 0.1	16.6 ± 0.2
30	DT13	NA	5.2 ± 0.1	NA	NA	17.5 ± 0.1	NA
31	HK26	28.8 ± 0.3	21.5 ± 0.1	12.3 ± 0.2	22 ± 0.1	NA	14.6 ± 0.1
32	HK23	NA	23.6 ± 0.5	NA	NA	5 ± 0.1	NA
33	SN28	5.1 ± 0.2	NA	NA	NA	7.5 ± 0.5	NA
34	SN23	15.1 ± 0.1	16 ± 0.1	NA	NA	20 ± 0.1	NA
35	HK38	NA	17.9 ± 0.1	11.6 ± 0.3	NA	NA	NA
36	HK37	17.1 ± 0.4	NA	NA	10 ± 0.1	NA	NA
37	HK17	23.7 ± 0.1	19.7 ± 0.1	25.2 ± 0.1	17.5 ± 0.1	16 ± 0.1	37 ± 0.6
38	HK9	3.8 ± 0.1	NA	NA	NA	NA	12.5 ± 0.1
39	SN26	5.9 ± 0.1	NA	NA	14.5 ± 0.1	NA	NA
40	HK19	17.9 ± 0.1	NA	6.6 ± 0.1	NA	NA	NA
41	SN24	NA	9 ± 0.1	NA	NA	10 ± 0.1	12 ± 0.1
42	HK30	12.8 ± 0.1	19.5 ± 0.7	NA	NA	NA	NA
43	DT9	3.5 ± 0.2	NA	NA	10.5 ± 0.1	NA	NA
44	HK32	31.6 ± 0.5	NA	NA	33.3 ± 0.2	14.6 ± 0.1	27.5 ± 0.3
45	HK2	23.1 ± 0.2	36.8 ± 0.3	NA	NA	NA	NA
46	KH18	NA	22.5 ± 0.1	NA	17.5 ± 0.3	NA	15 ± 0.1
47	HK29	29.3 ± 0.2	12.1 ± 0.2	NA	NA	NA	NA
48	TT19	30.3 ± 0.2	31.5 ± 0.1	18.5 ± 0.3	27.5 ± 0.1	25 ± 0.1	23.3 ± 0.2
49	TG1	42.6 ± 0.2	32 ± 0.1	32.5 ± 0.3	34 ± 0.1	28.5 ± 0.1	43.2 ± 0.1
50	DT23	5.1 ± 0.1	NA	4.3 ± 0.1	NA	NA	NA

*NA* No Activity

^a^Growth inhibition values are given as mean ± SD (*n* = 3)

### Chitinase production analysis

The 50 antagonistic isolates were subjected to the extracellular chitinase production and out of which 12 (24%) showed the most promising chitinase production. The chitinase producing isolates were further detected by PCR amplification of bacterial chitinase gene (Fig. S2). The sequencing of partial chitinase gene sequences was then translated to amino acid sequences for identification of their responsible chitinase gene and structures by using PDB protein databank. The amino acid sequences of the 8 isolates i.e., HK26, HK28, HK32, HK21, TG1, HK17, HK36 and KH49 showed 99% similarity with the ChiA gene of the *Serratia marcescens.* This ChiA gene is important in the chitin hydrolysis [3]. The other 4 isolates SN18, TG24, SN25 and TT19 showed 100% similarity at the amino acid level with ChiA74 gene of the *Bacillus thuringiensis*. The TIM-barrel/CID catalytic domain of ChiA74 of *Bacillus thuringiensis* involved in chitin hydrolysis [18] (Table 2).

**Table 2:** Amino acid sequence similarities of the chitinase gene of rhizobacteria with their activity and 3D structures in the PDB protein data bank

Strain Code	GenBank accession no.	PDB Top blast match	Similarity (%)	Gene Name	PDB ID	Activity	Structure	Reference
HK26	KY172957	*Serratia marcescens*	99%	ChiA	1RD6	*Serratia marcescens* chiA serves during chitin hydrolysis		Aronson et al., 2006 [3]
HK28	KY172958							
HK32	KY172959							
HK21	KY172956							
TG1	KY273607							
HK17	KY112753							
HK36	KY288868							
KH49	KY172960							
SN18	KY27360	*Bacillus thuringiensis*	100%	ChiA74	6BT9	TIM-barrel/CID catalytic domain of ChiA74 of *Bacillus thuringiensis* harbors the conserved motif in most family 18 chitinase involved in chitin hydrolysis		Juárez-Hernández et al., 2019 [18]
TG24	KY273608							
SN25	KY273606							
TT19	KY312499							

### ARDRA and BOX-PCR fingerprinting analysis

**Fig. 1:**
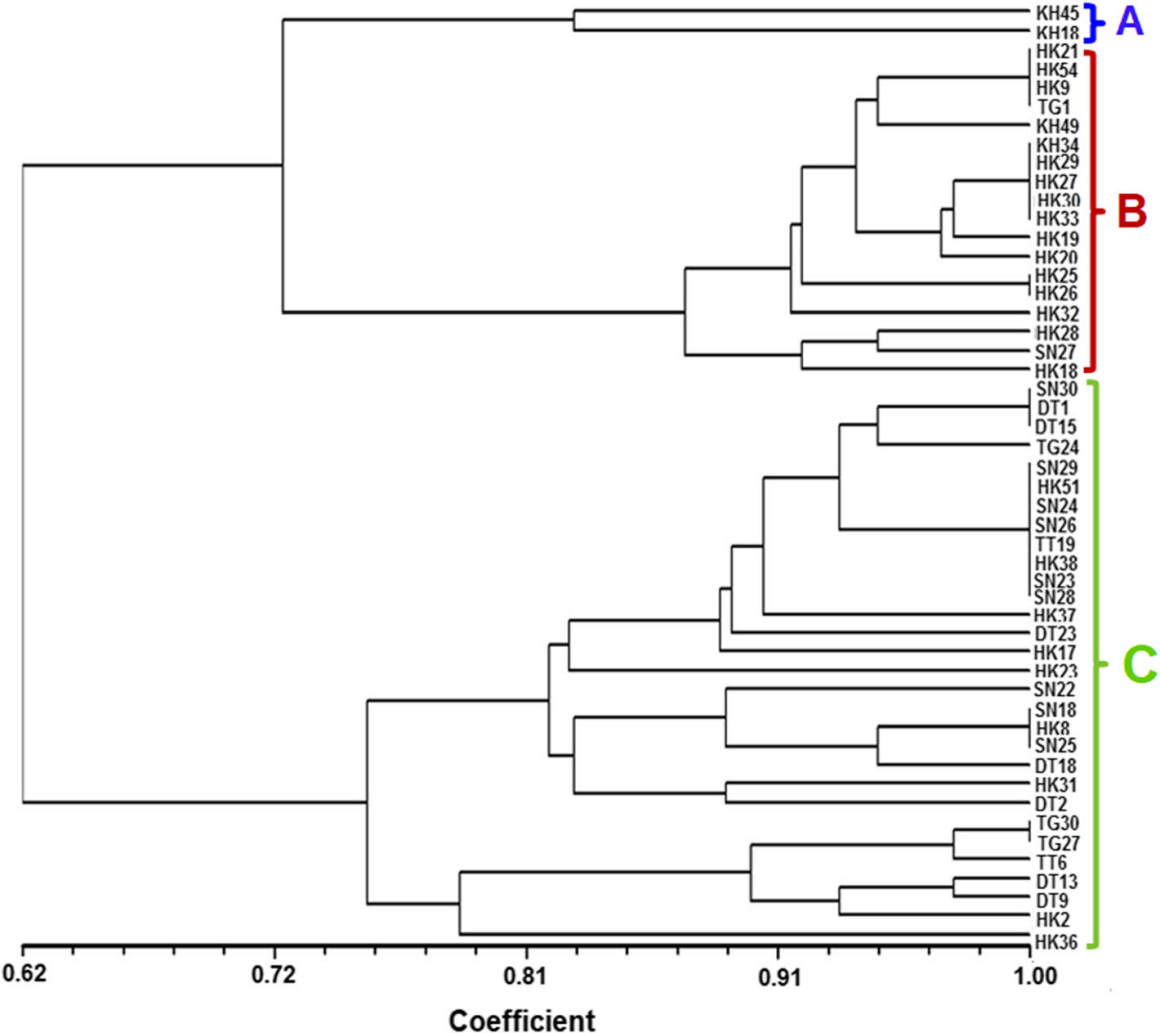
UPGMA dendrogram generated by Dice similarity coefficient index from ARDRA banding patterns of 50 antagonistic rhizobacteria using NTSYS 2.02. The scale on the x-axis refers to the similarity coefficient

**Fig. 2:**
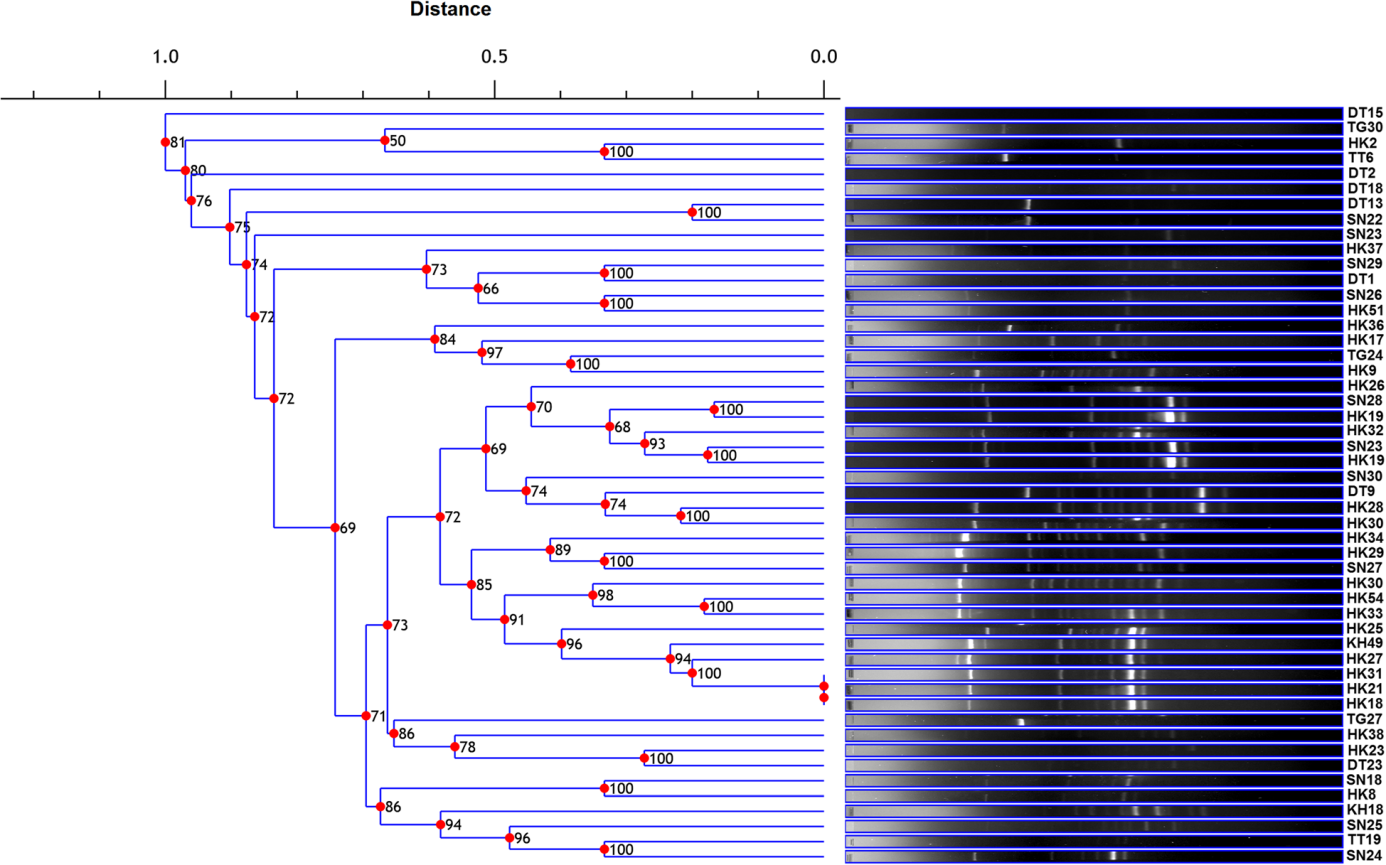
Dendrogram generated using Dice similarity coefficient index from BOX-PCR genomic fingerprints of 50 antagonistic rhizobacteria using Phoretix 1D software

The restriction digestion profile of selected 50 potential antagonistic rhizobacteria was analyzed by ARDRA fingerprinting using three different restriction enzymes HaeIII, MspI, HinfI. The digestion with these endonuclease restriction enzymes showed different banding patterns and dendrogram was constructed by analyzing these banding patterns. The dendrogram was analyzed by using the DICE similarity coefficient which is divided into three board distinct clusters A, B and C (Fig. 1). The genus *Bacillus* was found as a dominant bacterial genus which is mostly grouped in cluster C, cluster B is composed of both the genus *Serratia,* and *Enterobacter,* and A is composed of genus *Pseudomonas*. Similarly, the BOX-PCR fingerprinting was carried out and the dendrogram was constructed by using the DICE similarity coefficient considering the band size between 500 bp to 5 kb for scoring. The BOX-PCR generated a variation in the banding pattern of the isolates indicating the presence of different genotypes among the isolates (Fig. 2).

### Molecular identification and phylogenetic analysis

**Fig. 3:**
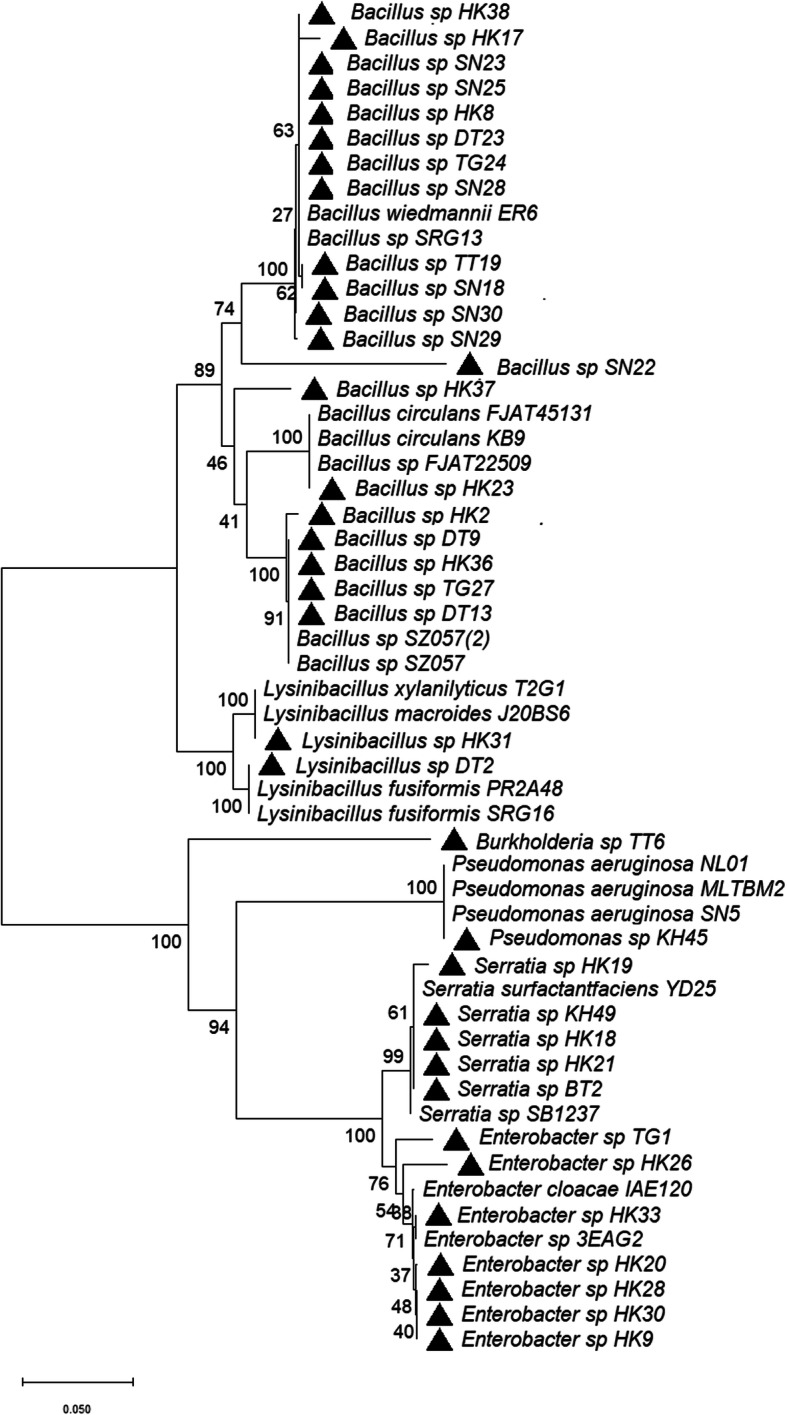
Phylogenetic tree showing the evolutionary relationship between selected potential antagonistic rhizobacteria and closest type strains based on the 16S rDNA sequences by NJ-method using Kimura-2 parameter model. The bar represents 0.05 substitutions per site, bootstrap values (*n* = 1000) are displayed

The 16S rDNA of 36 representative isolates were selected and identified with their closest homolog match using EzBioCloud 16S database. The 16S rDNA molecular identification of the 36 representative isolates revealed that the genus *Bacillus* was the most dominant (*n* = 21, 58.3%); followed by *Enterobacter* (*n* = 8, 22.2%); *Serratia* (*n* = 3, 8.3%); *Lysinibacillus* (*n* = 2, 5.5%); *Pseudomonas* (*n* = 1, 2.7%); and *Burkholderia* (*n* = 1, 2.7%). The phylogenetic tree was constructed based on neighbour-joining (NJ) method (Fig. 3). Moreover, the representative ribotypes identified from the rhizosphere soil of Assam tea estates, their closest sequence similarity and origin were described in Table 3.

**Table 3:** Molecular identification of 16S rRNA gene of representative antagonistic rhizobacteria with their sequence accession numbers and sample collection site from different commercial tea estates of Assam, India

Sl. No.	Isolate Code	GenBank accession no.	Base pair length	Top blast match with accession no. (EzBioCloud 16S Database)	Similarity (%)	Sampling site
1	HK2	KX986582	1417	*Bacillus safensis* FO-36b ASJD01000027	100	Hatikhuli tea estate
2	HK8	KX986583	1407	*Bacillus mobilis* 0711P9-1 MACF01000036	100	Hatikhuli tea estate
3	HK9	KX986584	1410	*Enterobacter chuandaensis* 090028 MK049966	99.7	Hatikhuli tea estate
4	HK17	KX986585	1350	*Bacillus cereus* ATCC 14579 AE016877	98.7	Hatikhuli tea estate
5	HK18	KX986586	1410	*Serratia marcescens* KRED AB061685	99.8	Hatikhuli tea estate
6	HK19	KX986597	747	*Serratia marcescens* SmUNAM836 CP012685	98.9	Hatikhuli tea estate
7	HK20	KX986598	763	*Enterobacter sichuanensis* WCHECl1597 POVL01000141	99.5	Hatikhuli tea estate
8	HK21	KX986587	1418	*Serratia marcescens* ATCC 13880 JMPQ01000005	99.3	Hatikhuli tea estate
9	HK23	KX986588	1410	*Bacillus circulans* ATCC 4513 AY724690	99.7	Hatikhuli tea estate
10	HK26	KX986599	649	*Enterobacter cloacae* LMG 2683 Z96079	97.4	Hatikhuli tea estate
11	HK28	KX986589	1405	*Enterobacter chuandaensis* 090028 MK049966	99.9	Hatikhuli tea estate
12	HK30	KX986600	763	*Enterobacter ludwigiio* EN-119 JTLO01000001	99.5	Hatikhuli tea estate
13	HK31	KX986601	739	*Lysinibacillus xylanilyticus* DSM 23493 LFXJ01000007	99.2	Hatikhuli tea estate
14	HK32	KX986590	734	*Enterobacter bugandensis* EB-247 FYBI01000003	99.5	Hatikhuli tea estate
15	HK33	KX986602	816	*Enterobacter sichuanensis* WCHECl1597 POVL01000141	99.8	Hatikhuli tea estate
16	HK36	KX986603	751	*Bacillus zhangzhouensis* DW5–4 JOTP01000061	99.1	Hatikhuli tea estate
17	HK37	KX986604	881	*Bacillus aryabhattai* B8W22 EF114313	100	Hatikhuli tea estate
18	HK38	KX986605	813	*Bacillus cereus* ATCC 14579 AE016877	99.5	Hatikhuli tea estate
19	DT2	KX986595	777	*Lysinibacillus fusiformis* NBRC 15717 AB271743	99.9	Difaloo tea estate
20	DT9	KX986579	1413	*Bacillus xiamenensis* HYC-10 AMSH01000114	99.9	Difaloo tea estate
21	DT13	KX986580	1414	*Bacillus pumilus* ATCC 7061 ABRX01000007	99.3	Difaloo tea estate
22	DT18	KX986581	837	*Bacillus toyonensis* BCT-7112 CP006863	91.5	Difaloo tea estate
23	DT23	KX986596	811	*Bacillus paramycoides* NH24A2 MAOI01000012	99.9	Difaloo tea estate
24	SN18	KX986607	731	*Bacillus proteolyticus* MACH01000033	99.9	Sonapur tea estate
25	SN22	KX986591	1412	*Bacillus marisflavi* JCM 11544 LGUE01000011	97.2	Sonapur tea estate
26	SN23	KX986608	782	*Bacillus nitratireducens* 4049 KJ812430	99.6	Sonapur tea estate
27	SN25	KX986609	794	*Bacillus paranthracis* Mn5 MACE01000012	100	Sonapur tea estate
28	SN28	KX986592	1402	*Bacillus wiedmannii* FSL W8–0169 LOBC01000053	100	Sonapur tea estate
29	SN29	KJ767523	1336	*Bacillus pseudomycoides* DSM 12442 ACMX01000133	99.6	Sonapur tea estate
30	SN30	KX986593	1392	*Bacillus* sp. AFS092012 NVOR01000041	99.9	Sonapur tea estate
31	TG1	KJ767522	1341	*Enterobacter lignolyticus* SCF1 CP002272	99.6	Tocklai tea growing area
32	TG24	KX986594	1410	*Bacillus albus* N35-10-2 MAOE01000087	100	Tocklai tea growing area
33	TG27	KX986610	823	*Bacillus altitudinis* 41KF2b ASJC01000029	100	Tocklai tea growing area
34	TT6	KJ767524	1315	*Burkholderia ambifaria* AMMD CP000442	99.3	Teok tea estate
35	TT19	KX986611	1415	*Bacillus proteolyticus* TD42 MACH01000033	99.9	Teok tea estate
36	KH45	KJ767521	1284	*Pseudomonas aeruginosa* JCM 5962 BAMA01000316	99.7	Khetri tea estate

## Discussion

There are diverse microbial communities that resides in the rhizosphere zone of plants and they are studied for their functional roles in the soil, plants and biogeochemical cycles. The aim of the present study was to study diverse autochthonous culturable antagonistic rhizobacterial communities present in the commercial tea estates of Assam, North-eastern part of India which is situated in the Indo-Burma mega-biodiversity hotspot. The presence of biodiversity hotspot in the region fuelled this study to search for the potential microbial isolates for the diverse biotechnological applications.

In this study, a total of 217 isolates were isolated from tea rhizosphere soil of six different commercial tea estates of Assam, India. The rhizosphere soil is a niche for diverse beneficial microorganisms and one of the most intensive plant microbe interactions zone. The pH of the soil range was found to be from 4.1 to 5.2 which indicating to the soil is acidic in nature. Studies on tea plantations have shown that soil becomes low pH under tea plantation and also acidification determined the age and use of fertilizer in tea plantation [1, 2].

The antagonistic activity of 217 isolates was carried out against six fungal phytopathogens and out of which 50 isolates showed potential antagonistic activity. These 50 isolates were considered as elite isolates for further investigation. Moreover, it was also observed that out of these 50 isolates, 16 (32%) isolates exhibited antifungal activity against at least four test pathogens and 6 (12%) isolates exhibited the antagonistic activity against all the test fungal pathogens. Plant associated rhizosphere microbial communities are considered as a crucial first line defence for disease suppression in plants. It was also observed that the root exudates of the infected plants are more attractive than uninfected plants to harbour the model strain *Pseudomonas protegens* CHA0 for production of disease suppressive antimicrobial metabolites [7]. The in vitro antifungal activity of *Bacillus subtilis* producing lipopeptides was tested against apple scab causing ascomycete fungi *Venturia inaequalis* [6]. Another study on gram-positive *B. subtilis* 30VD-1 has shown very potential antagonistic activity against ascomycete fungi *Fusarium* sp. plant pathogen [19]. Similarly, gram negative *Enterobacter* sp. BNM 0357 strain isolated from rhizosphere soil has been demonstrated to inhibit up to 35% of the mycelial growth and spore germination of *Fusarium solani* [31]. In the present study, 2 g negative strains *Enterobacter* sp. HK28 and TG1 and 4 strains of gram-positive *Bacillus* sp. SN18, SN25, HK17 and TT19 have demonstrated board spectrum antifungal activity against five ascomycetes fungi i.e., *P. theae* (ITCC 6599), *C. eragrostidis* (ITCC 6429), *G. cingulata* (MTCC 2033), *F. oxysporum* (MTCC 284), *N. sphaerica* (KJ767520) and one basidiomycete fungal pathogen *R. solani* (MTCC 4633). This board spectrum antifungal result suggests that the rhizosphere soil of plant is a good source of potential antagonistic rhizobacteria to the encounter the plant pathogens.

Chitinases are very useful enzymes for different applications due to its key role in the degradation of crystalline polysaccharides. They catalyse the hydrolysis of β-1, 4-linkages in chitin which exert a direct inhibitory effect on the hyphal growth of fungal pathogens [27]. In our study, 12 isolates out of 50 potential antagonistic isolates showed the positive for extracellular chitinase activity in plate chitinase enzyme assay and PCR based chitinase gene detection. Interestingly, the 6 isolates which showed board spectrum antagonistic activity against the fungal pathogens, also exhibited the chitinase activity and presence of chitinase gene. The analysis of the chitinase gene of 12 isolates at amino acid level with their 3D structures in PDB protein databank revealed that the isolates HK26, HK28, HK32, HK21, TG1, HK17, HK36 and KH49 showed 99% similarity with the ChiA gene of the *Serratia marcescens* and isolates SN18, TG24, SN25 and TT19 showed 100% similarity at the amino acid level with ChiA74 gene of the *Bacillus thuringiensis*. A study on expression of different chitinase genes (ChiA, ChiB, ChiC and ChiD) was conducted at the transcriptional level and found that the presence of chitin strongly induced the ChiA gene for hydrolysis of chitin followed by others three chitinase genes [26]. Another study on combination of purified chitinases (ChiA, ChiB and ChiC) from *S. marcescens* strain CFFSUR-B2 isolate significantly suppressed the germination and germ tube growth of *Mycosphaerella fijiensis* causes black Sigatoka disease of banana [15]. The chitinase enzyme isolated from *Serratia marcescens* B4A strain exhibited strong antagonistic activity against *Rhizoctonia solani, Bipolaris* sp., *Alternaria raphanin,* and *Alternaria brassicicola* [39]. Similarly, the *Bacillus thuringiensis* C25 isolate having cell wall degrading enzymes such as protease, β-1,3-glucanase and chitinase were also reported to inhibit the mycelial growth, and suppression of sclerotia formation and germination of two major sclerotia phytopathogens [32]. The presence of chitinase gene in the 12 isolates of the present study strongly implies the exhibition of antifungal activity was due to the presence of chitinase enzyme. However, the absence of chitinase genes does not solely determine the antagonistic activity of the isolates because the other mechanisms or bioactive agents may be involved in the production of antifungal activity.

The ARDRA and BOX-PCR molecular genotyping are robust and most widely used for the classification and identification of culturable microbial communities at the level of genus and species [20]. These molecular tools are suitable and convenient for species-specific fingerprint and phylogenetic analysis [24]. The genetic diversity of the 50 antagonistic rhizobacteria through ARDRA using three different restriction enzymes HaeIII, MspI, HinfI and BOX-PCR fingerprinting revealed significant differences among the isolates. However, the bacterial diversity and richness in soil are largely differed by ecosystem type and pH of the soil or other edaphic variables [13]. The study of microbial diversity and 16S rDNA sequencing of acidic soil of tea rhizosphere comprised of genus *Bacillus, Enterobacter, Serratia, Lysinibacillus, Pseudomonas* and *Burkholderia*. Based on the molecular fingerprinting and chitinase gene analysis, 36 representative ribotypes were selected for diversity analysis. The analysis of these ribotypes using EzBioCloud 16S database revealed that 21 (58.3%) isolates belonged to the genus *Bacillus*, 8 (22.2%) *Enterobacter*, 3 (8.3%) *Serratia*, 2 (5.5%) *Lysinibacillus* and 1 (2.7%) isolate to each of the genus *Pseudomonas* and *Burkholderia*. The gram-positive *Bacillus* and *Bacillus* like genus dominance was also reported by the previous studies on soil [5, 9, 21, 23]. From the present study, it was established that the genus *Bacillus* is also the most predominant group of bacteria in the tea rhizosphere soil of Assam, India.

## Conclusion

The rhizosphere soil is a source of intensive microbial communities represents it beneficial effects on the overall health of the plant and soil. There is cross-talk between the plants and microbes in this zone to help each other in different adverse conditions. The diverse antagonistic bacterial communities present in the rhizosphere indicating its devoted service to the ecosystem management and protection of plants from various pathogenic microorganisms. Therefore, the findings in the present study revealed that antagonistic rhizobacteria isolated from the tea rhizosphere soil can be a valuable source for application in agriculture and industrial prospective.

## Methods

### Site description, sampling and isolation of bacteria

Assam state is located in the Northeast region of India and extending from 89^0^42′ E to 96^0^ E longitude and 24^0^8′ N to 28^0^2′ N latitude with an area of 78,438 km^2^. With the tropical monsoon rainforest climate, Assam is a temperate region and experiences heavy rainfall and humidity. The tea plantations are one of the most economically important plants grown in Assam. Because of its long growing season and generous rainfall, Assam is one of the most prolific tea-producing regions in the world. The state is cultivated in over 304,400 ha area with an annual production of 629.05 million kg which contributes more than 50% of the overall annual tea production of India.

The rhizosphere soil samples of tea plants were collected from 5 to 30 cm depth soil in sterile bags and transported immediately to the laboratory in icebox. These soil samples were collected from six different tea estates located in Assam, India i.e. Sonapur tea estate (26^0^06′56.40′′N 91^0^58′33.18′′E), Khetri tea estate (26^0^06′53.81′′N 92^0^05′27.74′′E), Toklai tea growing area (26^0^45′18.40′′N 94^0^13′16.92′′E), Difaloo tea estate (26^0^36′29.41′′N 93^0^35′03.96′′E), Teok Tata tea estate (26^0^36′29.41′′N 94^0^25′42.59′′E) and Hatikhuli tea estate (26^0^34′55.94′′N 93^0^24′43.15′′E). These sampling sites was also previously described in our research article which was the part of our undergoing tea rhizosphere soil research work [8]. The bacteria were isolated from rhizosphere soil by using the serial dilution method. For this, 1 g of the soil was suspended in 9 ml of saline solution (i.e., 0.9% NaCl) and kept in shaking condition for 30–45 min at 200 rpm and 30 °C. The soil suspension was then serially diluted up to 10^− 6^ and 100 μl from each dilution was evenly spread over the surface of four different isolation media agar plates i.e., Nutrient agar, *Pseudomonas* isolation agar, *Azotobacter* agar and *Azospirillum* agar (HiMedia, India). The plates were then incubated for 12–24 h at 30 °C and bacterial colonies that appeared on different media were selected based on their different colony morphology [8].

### In vitro antifungal assay

#### Test fungal pathogens

The six tea fungal pathogens were used for this study i.e., *Pestalotiopsis theae* (ITCC 6599), *Curvularia eragrostidis* (ITCC 6429), *Glomerella cingulata* (MTCC 2033), *Rhizoctonia solani* (MTCC 4633), *Fusarium oxysporum* (MTCC 284), and *Nigrospora sphaerica* (KJ767520). The fungal pathogens were obtained from the Microbial Type Culture Collection (MTCC), and the Institute of Microbial Technology, Chandigarh, India and Indian Type Culture Collection (ITCC), Indian Agricultural Research Institute, New Delhi, India. The *Nigrospora sphaerica* tea fungal pathogen was isolated, characterized and preserved at Institute of Advanced Study in Science & Technology, Guwahati, India.

#### In vitro screening of tea rhizobacteria for antifungal activity

The isolated rhizobacteria were subjected to evaluate their antagonistic potential against the selected tea fungal pathogens. The bacterial broth cultures were adjusted to 1 × 10^8^ CFU/ml and a loopful of bacterial cultures was streaked equidistantly on the edge of the PDA plates. The 5 mm agar plug of the test fungal mycelium previously grown on the PDA plate was placed at the center of the test plate between the bacterial streaked lines. The plates were incubated at 28 ± 2 °C for 5 days. The control plates were prepared with the fungal agar plug without the bacterial streaks. The antagonistic activity was evaluated by comparing the fungal mycelial diameter on control and test plates and the percentage of inhibition was calculated by using the formula C-T/C × 100, where, C is the fungal mycelial diameter on the control plate and T is the fungal mycelial diameter on the test plate [9, 10]. The experiments were performed in triplicates.

### Chitinase production

#### Preparation of colloidal chitin

For the preparation of colloidal chitin, 5 g of shrimp shells chitin (Sigma, USA) was slowly added into 100 ml of cold 0.25 N HCl with vigorous stirring and kept overnight at 4 °C. The mixture was filtered through the filter paper into 200 ml ice cold ethanol at 4 °C with rapid stirring. The chitin suspension was centrifuged at 10,000×g for 20 min and the resultant chitin pellet was washed repeatedly with sterile distilled water until the pH became neutral [28]. The final concentration was adjusted to 10 mg/ml.

#### Chitinase production and PCR amplification of chitinase gene

The bacterial isolates were evaluated for the hydrolysis of chitin by using spot inoculation method on MS media containing 1% chitin (v/v). After pouring the chitin containing medium into the petri plates, the bacterial inoculums were spotted on the plate and incubated for 48 h to observe the zone of clearance [12].

The genomic DNA was extracted by using QIAamp DNA mini kit (Qiagen, India), and the presence of bacterial chitinase gene were screened by using degenerate primes GA1F and GA1R [35]. The 10 μl of PCR reaction volume comprised of 1 μl of 10 × Taq DNA buffer, 2.5 mM dNTP mix, 0.2 μM of primers, 1 U Taq polymerase and 1 μl of 10 ng concentration of template DNA. The amplifications were carried out in the proflex PCR system (Applied Biosystems, USA). The reaction was set as follows: an initial denaturation at 95 °C for 5 min, 35 cycles of 1 min at 95 °C, 30 s at 60 °C, 1 min at 72 °C followed by one cycle of 7 min at 72 °C for the final extension. The partially amplified chitinase genes were sequenced and translated to amino acid sequences using the ORF-Finder (https://www.ncbi.nlm.nih.gov/orffinder/). The resulting amino acid sequences were used as queries to search the related proteins in the PDB protein databank (PDB; http://www.rcsb.org/pdb/) using the BLAST algorithm based advanced sequence search with the default parameters.

#### Amplified ribosomal DNA restriction analysis (ARDRA)

The ARDRA technique is basically based on restriction endonuclease digestion of the amplified bacterial 16S rDNA. The genomic DNA extraction, 16S rDNA PCR amplification and purification of the PCR product was carried out as previously described [9]. For ARDRA analysis, 20 μl (50 ng) of 16S rDNA purified PCR products were digested by 1.5 U of three different restriction enzymes HaeIII, MspI, HinfI (New England Biolabs, UK) according to manufacturer’s instruction and incubated for 3 h at 37 °C. The resulting digested fragments together with 100 bp ladder (Merck Genei, India) were resolved by gel electrophoresis at 60 V on 2% agarose gels in 1 × TAE buffer containing 10 μg/ml of EtBr. The gel profile obtained were analyzed by considering, the character state “1” for clearly detected bands in the gel track and assigned “0” if it was absent or impossible to determine. The data matrix thus generated was calculated by Dice similarity coefficient. Each pairwise comparison was constructed from the similarity matrix by the unweighted pair group method with arithmetic mean (UPGMA) using DICE similarity coefficient and the TreePlot program in NTSYSpc 2.02e analysis package (Applied Biostatistics Inc., New York).

#### BOX-PCR fingerprinting

The fingerprinting of antagonistic bacterial isolates was performed by repetitive extragenic palindromic-PCR (rep-PCR). The rep-PCR was carried out by using the BOX-A1R primer (5′-CTACGGCAAGGCGACGCTGACG-3′) [24]. The PCR product obtained was subjected to electrophoresis with a 500 bp DNA ladder (Merck Genei, India) using 2% agarose gel in 1 × TAE buffer containing 10 μg/ml of EtBr. The generated fingerprints were further analyzed by hierarchical clustering using DICE similarity coefficient in Phoretix 1D Pro gel analysis software (TotalLab Ltd., Newcastle upon Tyne, England).

#### Molecular characterization and phylogenetic analysis

For the 16S rDNA identification, the rhizobacteria were selected based on the chitinase activity, ARDRA and BOX-PCR fingerprinting using the facility at Scigenom Labs Pvt. Ltd. (Cochin, India). The raw forward and reverse sequences obtained after sequencing of the isolates were analyzed by Sequence Scanner 2.0 software (Applied Biosystems) to filter out the low-quality base calls. The low-quality base calls were trimmed from both the sequences and aligned to remove the overlap regions. Then the contigs generated were assembled and screened for chimeras using DECIPHER software [36]. The 16S rDNA sequences generated after screening were identified by EzBioCloud 16S database [38] and submitted to the GenBank. The 16S rDNA gene sequences of antagonistic bacterial isolates along with their closest homology sequences retrieved from NCBI GeneBank were aligned by using multiple sequence alignment CLUSTAL W algorithm executed in MEGA X software [33]. These aligned sequences were used to construct the phylogenetic tree using neighbour-joining (NJ) method by MEGA 6 program and evolutionary distances were computed with the help of Kimura’s 2 parameter model [22]. Bootstrap analysis with 1000 replications using p-distance model was performed to estimate the confidence of a particular clade [11].

#### Sequences submitted to GenBank database

The 36 nucleotide sequences of 16S rDNA of rhizobacterial isolates from Assam tea estates were submitted in GenBank under NCBI accession no. KJ767521-KJ767524 and KX986579-KX986611. The 12 chitinase gene sequences were submitted under GenBank accession no. KY172956-60, KY273605-08, KY112753, KY288868, KY312499.

#### Data analysis

All experiments were performed in triplicates to calculate the mean values and data were expressed as mean ± standard deviation. The isolates showing antagonistic activity against the different fungal pathogens were represented as Venn diagram using the multiple dataset analysis features of VENNTURE software.

## Supplementary Information

**Additional file 1 **: **Fig. S1**. Venn diagram of antifungal assay showing distribution of 50 antagonistic rhizobacteria into six profiles which are representing the 6 test fungal pathogens. (*6 isolates showed antagonistic activity against all the test fungal pathogens). **Fig. S2**. PCR amplification of chitinase gene of 12 potential rhizobacteria. (M-100bp ladder, 1–12 chitinase positive rhizobacteria strains i.e., HK26, HK28, HK32, HK21, TG1, HK17, HK36, KH49, SN18, TG24, SN25 and TT19 respectively).

## Data Availability

The sequence analysis during the current study are available in the in GenBank repository under NCBI accession no. KJ767521-KJ767524, KX986579-KX986611, KY172956-60, KY273605-08, KY112753, KY288868, KY312499.
