# Supplementary material for: Diversity of culturable bacteria endowed with antifungal metabolites biosynthetic characteristics associated with tea rhizosphere soil of Assam, India
Source: BMC Microbiol. 2021 Jul 18;21:216. doi: 10.1186/s12866-021-02278-z (PMC8286567; doi:10.1186/s12866-021-02278-z)
Supplement: Supplementary file 1 — Additional file 1 : Fig. S1. Venn diagram of antifungal assay showing distribution of 50 antagonistic rhizobacteria into six profiles which are representing the 6 test fungal pathogens. (*6 isolates showed antagonistic activity against all the test fungal pathogens). Fig. S2. PCR amplification of chitinase gene of 12 potential rhizobacteria. (M-100bp ladder, 1–12 chitinase positive rhizobacteria strains i.e., HK26, HK28, HK32, HK21, TG1, HK17, HK36, KH49, SN18, TG24, SN25 and TT19 respectively). [file 12866_2021_2278_MOESM1_ESM.docx]

**Supplementary Materials**

Jintu Dutta^1,2^ Debajit Thakur^1^

^1^Microbial Biotechnology Laboratory, Life Sciences Division, Institute of Advanced Study in Science and Technology, Guwahati, Assam, India

^2^Centre for the Environment, Indian Institute of Technology Guwahati, Assam, India (Present address)

Corresponding author: Debajit Thakur

debajitthakur@iasst.gov.in

Contact: +91-9706939610

Microbial Biotechnology Laboratory, Life Sciences Division, Institute of Advanced Study in Science and Technology, Guwahati, Assam, India


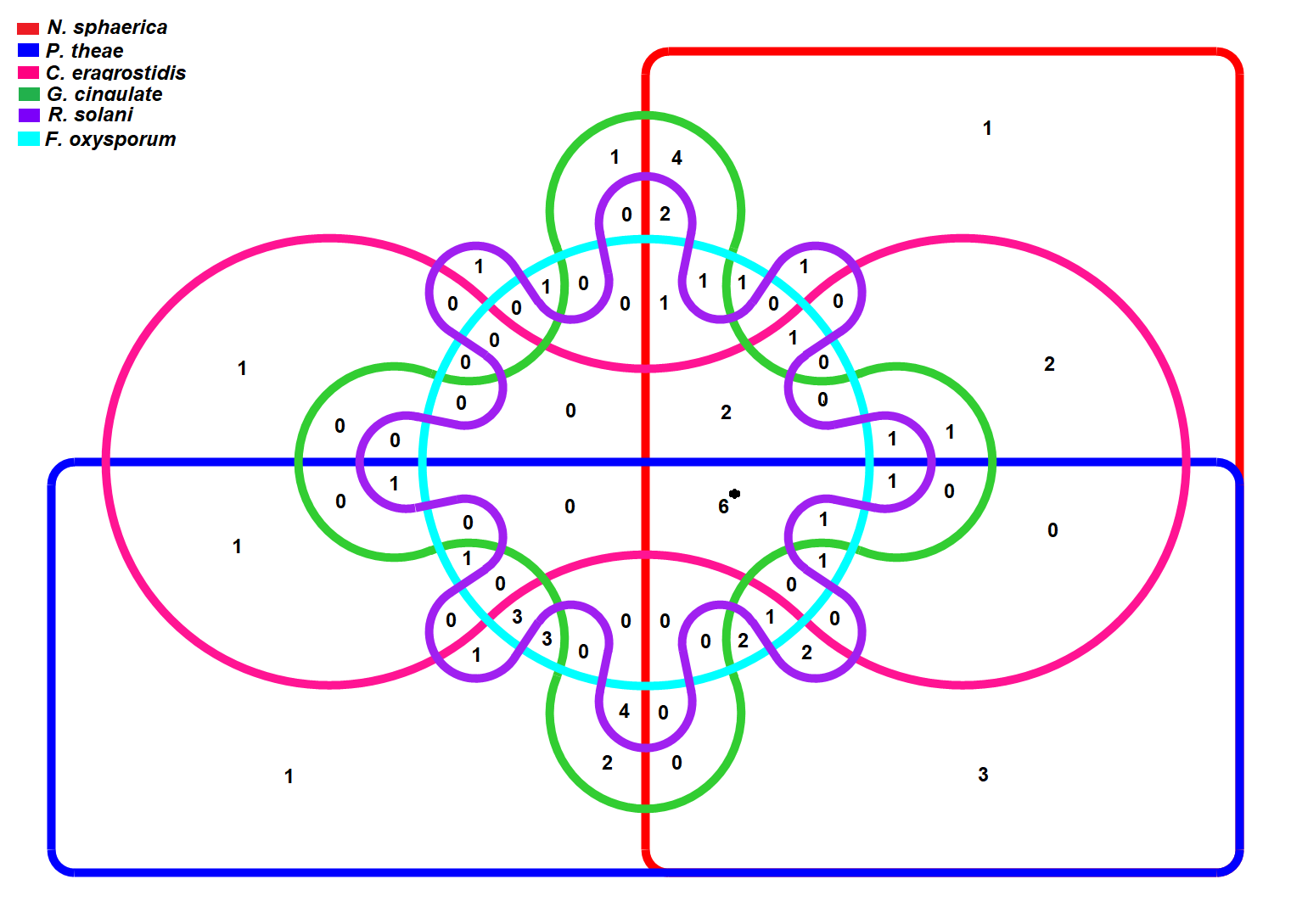


**Fig. S1** Venn diagram of antifungal assay showing distribution of 50 antagonistic rhizobacteria into six profiles which are representing the 6 test fungal pathogens. (*6 isolates showed antagonistic activity against all the test fungal pathogens)


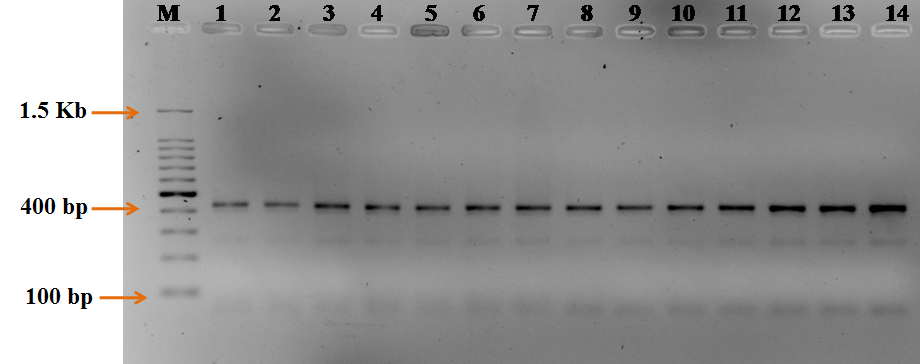


**Fig. S2** PCR amplification of chitinase gene of 12 potential rhizobacteria. (M-100bp ladder, 1–12 chitinase positive rhizobacteria strains i.e., HK26, HK28, HK32, HK21, TG1, HK17, HK36, KH49, SN18, TG24, SN25 and TT19 respectively).
